# Study on salvianolic acid B in the reduction of epidural fibrosis in laminectomy rats

**DOI:** 10.1186/1471-2474-15-337

**Published:** 2014-10-07

**Authors:** Feng Chen, Zhenbo Zuo, Kai Wang, Chengdong Zhang, Haifeng Gong, Fagang Ye, Aiyu Ji, Hao Tao

**Affiliations:** Department of Trauma, Affiliated Hospital of Qingdao University, Qingdao, P.R. China

**Keywords:** Epidural fibrosis, Salvianolic acid B, Laminectomy, Rat

## Abstract

**Background:**

Epidural fibrosis (EF) is a common complication after laminectomy. Salvianolic acid B (Sal B) is a major bioactive component of a traditional Chinese medical agent, *Salvia miltiorrhiza*, which has shown anti-inflammatory, anti-fibrotic and anti-proliferative properties. The object of this study was to investigate the effect of Sal B on the prevention of epidural fibrosis in laminectomy rats.

**Methods:**

A controlled double-blinded study was conducted in sixty healthy adult Wistar rats that underwent laminectomy at the L1-L2 levels. The rats were randomly divided into 3 groups of 20: (1) Sal B treatment group; (2) Vehicle group; (3) Sham group (laminectomy without treatment). All rats were sacrificed 4 weeks post-operatively. The extent of epidural fibrosis, fibroblast proliferation and the expression of vascular endothelial growth factor (VEGF) and inflammatory factors were analyzed.

**Results:**

The recovery of all rats was uneventful. In the laminectomy sites treated with Sal B, the dura mater showed no adhesion. Collagen deposition was significantly lower in the Sal B group than the other two groups. In addition, both fibroblast and inflammatory cell counting in the laminectomy sites treated with Sal B showed better grades than the other two groups. The expression of VEGF and inflammatory factors in operative sites also suggested better results in the Sal B group than the other two groups.

**Conclusions:**

Sal B inhibits fibroblast proliferation, blood vessel regeneration, and inflammatory factor expression. Thus, Sal B is able to prevent epidural scar adhesion in post-laminectomy rats.

**Electronic supplementary material:**

The online version of this article (doi:10.1186/1471-2474-15-337) contains supplementary material, which is available to authorized users.

## Background

Failed back surgery syndrome (FBSS) is gaining attention from both lumbar surgeons and lumbar laminectomy patients. FBSS occurs in 8-40% of patients who undergo lumbar disc surgery[1]. Recurrent persistent low back pain and chronic nerve radicular are the main characteristics for FBSS[2]. Epidural fibrosis (EF) is widely accepted to be the main contributor to FBSS[3–5].

EF, as a scar tissue adjacent to the dura mater following lumbar laminectomy, can lead to extensive nerve roots and dural mater adhesions. Therefore, EF could cause restriction of nerve root mobility, dural compression and spinal canal stenosis[6].

Various attempts have been made to prevent EF, such as topical application of immunosuppressive agents[7], anti-inflammatory agents[8], calcium channel blockers[6], animal collagen membranes[9], modified meticulous surgery[10], and traditional Chinese medical agents, such as pseudo-ginseng and *Angelica sinensis*[5, 11]. Although some of them have achieved moderate success in animals, there is still no single agent or biomaterial that has reached the success of clinic application.

Salvianolic acid B (Sal B), a major bioactive component of the traditional Chinese medical agent, *Salvia miltiorrhiza*, is widely accepted for use against cardiovascular diseases[12]. Sal B is reported to exert anti-inflammatory and neuroprotective effects both in vivo and in vitro[13–15]. And with the advantages of little or no toxicity, Sal B is also able to alleviate liver fibrosis[16]. However, there have been no studies investigating the effects of Sal B on preventing EF.

In the present study in laminectomized rats, we evaluated the efficacy of Sal B in the prevention of EF. Specifically, macroscopic assessment, histological analysis, and measurement of inflammatory factors and hydroxyproline content were applied.

## Methods

### Subjects

A total of sixty adult healthy male Wistar rats (mean weight 250 g) were employed. The present research was approved by Qingdao University Medical College Medical Ethics Committee. In compliance with the principles of International Laboratory Animal Care and with the European Communities Council Directive (86/809 /EEC), animals were housed in the local laboratory under the conditions of 20 to 25°C room temperature, a 12 hour light–dark cycle and clean food and water ad libitum (Additional file1). All efforts were made to minimize animal numbers and suffering. The rats were housed for 10 days to adjust them to the environment pre-operatively. Animals were randomly divided into three groups (20 rats in each group): 1) Sal B treatment group (Sal B 30 mg/kg, diluted in saline); 2) Vehicle group (saline); 3) Sham group (laminectomy without treatment).

### Agents and antibodies

Sal B (purity > 98%) was purchased from the National Institute for the Control of Pharmaceutical and Biological Products (Beijing, China). Cal-EX II solution for decalcification and dehydration was purchased from Thermo Fisher Scientific (Waltham, MA, USA). β-Dimethylaminobenzaldehyde was purchased from Sigma-Aldrich (St. Louis, MO, USA). Reverse Transcriptase was purchased from Promega (Madison MA, USA). Primary antibody and secondary antibodies were purchased from abcam (Cambridge, UK).

### Surgery

The rat laminectomy model was created as previously reported[4–7]. The surgery was performed under sterile conditions with basic surgical tools. Anesthetization was performed by intra-peritoneal injection of 10% chloral hydrate (0.3 ml/100 g body weight). All the rats were numbered individually after being restrained on a warm pad in the prone position. The fur of each rat was shaved around L1 and L2 and the exposed skin was sterilized. After that L1-L2 total laminectomy was performed with a micro-rongeur. Close attention was paid not to traumatize the dura and the nerve roots. After complete hemostasis with saline, the wound site was surgically closed.

### Salvianolic acid B administration

In the Sal B treatment group, salvianolic acid B (30 mg/kg, diluted in saline) was administered intragastrically[17]. In the Vehicle group, the same volume of saline was given in the same way. In the Sham group, no special treatment was performed post-laminectomy.

### Macroscopic assessment of EF

Macroscopic assessment was performed four weeks post-laminectomy. Five rats were randomly selected from each group and anesthetized. Then, the surgical sites were reopened. The epidural adhesion was evaluated by assistants in a double-blinded manner based on the Rydell classification[4] (grade 0 = epidural scar tissue was not adherent to the dura mater; grade 1 = epidural scar tissue was adherent to the dura mater, but easily dissected; grade 2 = epidural scar tissue was adherent to the dura mater, and it was difficult to dissect without disrupting the dura matter; grade 3 = epidural scar tissue was firmly adherent to the dura mater and could not be dissected).

### Determination of Hydroxyproline content (HPC) analysis

HPC analysis was performed according to a previous studies four weeks post-operatively[6, 7]. Six rats in each group were selected. The scar tissue, approximately 5 mg wet weight, was collected around the laminectomy site. The samples were rinsed, homogenized, centrifuged, and hydrolyzed. One milliliter of hydroxyproline developer (β–dimethylaminobenzaldehyde solution) was added to the samples and the standards. The absorbance at 550 nm was read with a spectrophotometer. HPC per milligram of scar tissue was calculated.

### Histological analysis

Histological analysis was performed four weeks post-laminectomy on five rats in each group. The whole L1-L2 vertebral column, including muscles and epidural scar tissue, was harvested and fixed in 10% phosphate-buffered formaldehyde solution. Decalcification and dehydration were performed with Cal-Ex II solution for 3 days. Five-micrometer axial sections of the laminectomy site were cut and stained with hematoxylin-eosin (H&E) and Masson’s trichrome.

The epidural scar adhesion was evaluated under the light microscope (Olympus ix-71). The number of fibroblasts and inflammatory cells were calculated based on a previous study[5]: grade 1, fewer than 100 fibroblasts/inflammatory cells per × 400 field; grade 2, 100 to 150 fibroblasts/inflammatory cells per × 400 field; grade 3, more than 150 fibroblasts/inflammatory cells per × 400 field. Three different counting areas were selected at the middle and at the margins of the laminectomy sites. The cells were counted and their mean was calculated.

To further quantify the density of blood vessels in each scar tissue, the VEGF immunohistochemistry was performed with the monoclonal anti-VEGF antibody, and the density of VEGF was evaluated. The fields to be counted for VEGF expression were chosen at 400× magnification from well-labeled areas. Staining of VEGF was assessed with a semi-quantitative grading system that reflected the intensity of staining within 20 randomly chosen blood vessels in each scar: (0) no staining; (1) weak staining; (2) moderate staining; and (3) strong staining. Each scar was then given an overall modified H-score ((number of vessels that scored 0) × 0 + (number of vessels that scored 1) × 1 + (number of vessels that scored 2) × 2 + (number of vessels that scored 3) × 3)[18, 19].

### Analysis of IL-6 and TGF-β1 concentrations

The mRNA analyses of IL-6 and TGF-β1 were performed four weeks post-operatively. Six rats in each group were selected. The scar tissues were collected from the laminectomy sites, and the total RNA was extracted. The RNA (2 μg) was transcribed into cDNA with AMV Reverse Transcriptase. Quantitative real-time PCR (qPCR) was performed based on a previous study using the Bio-Rad MYIQ2 (USA)[4]. Primers used are shown in Table 1. GAPDH amplification was employed as an internal control.

**Table 1:** **Primer sequences**

TGF-β1 (148 bp)	forward, 5′-GCCCTGCCCCTACATTTGG-3′
	reverse, 5′-CTTGCGACCCACGTAGTAGACGAT-3′;
IL-6 (131 bp)	forward, 5′-ACCCCAACTTCCAATGCTCT-3′
	reverse, 5′-TGCCGAGTAGACCTCATAGTGACC-3′
GAPDH (169 bp)	forward, 5′-TCACCACCATGGAGAAGGC-3′
	reverse, 5′-GCTAAGCAGTTGGTGGTGCA-3′

### Statistical analysis

The statistical analysis was performed with SPSS 13.0 statistical package (SPSS Inc., Chicago, IL, USA). Data are expressed as the mean ± standard deviation. Single-factor analysis of variance (ANOVA) and the q-test were applied to evaluate three independent samples. Statistical significance was assumed at p < 0.05.

## Results

### Macroscopic assessment of epidural scar adhesion

The recovery of all rats was uneventful. All rats showed no sign of wound infection, neurological deficit or disturbance of wound healing.

In the Sal B group, soft or weak fibrous adhesion was observed. In the Vehicle and Sham groups, severe epidural adhesions were seen. The dissection of epidural scar tissue would have led to serious bleeding and the risk of dura mater disruption or nerve root injury. It was impossible to re-expose the dura mater completely. The grades of epidural scar adhesion were evaluated according to the Rydell standard (Table 2).

**Table 2:** **Grades of epidural scar adhesion in rats, according to the Rydell standard**

Group	Grade			
	0	1	2	3
Sham	5	0	0	0
Vehicle	0	0	1	4
Sal B	4	1	0	0

### Hydroxyproline content (HPC)

**Figure 1:**
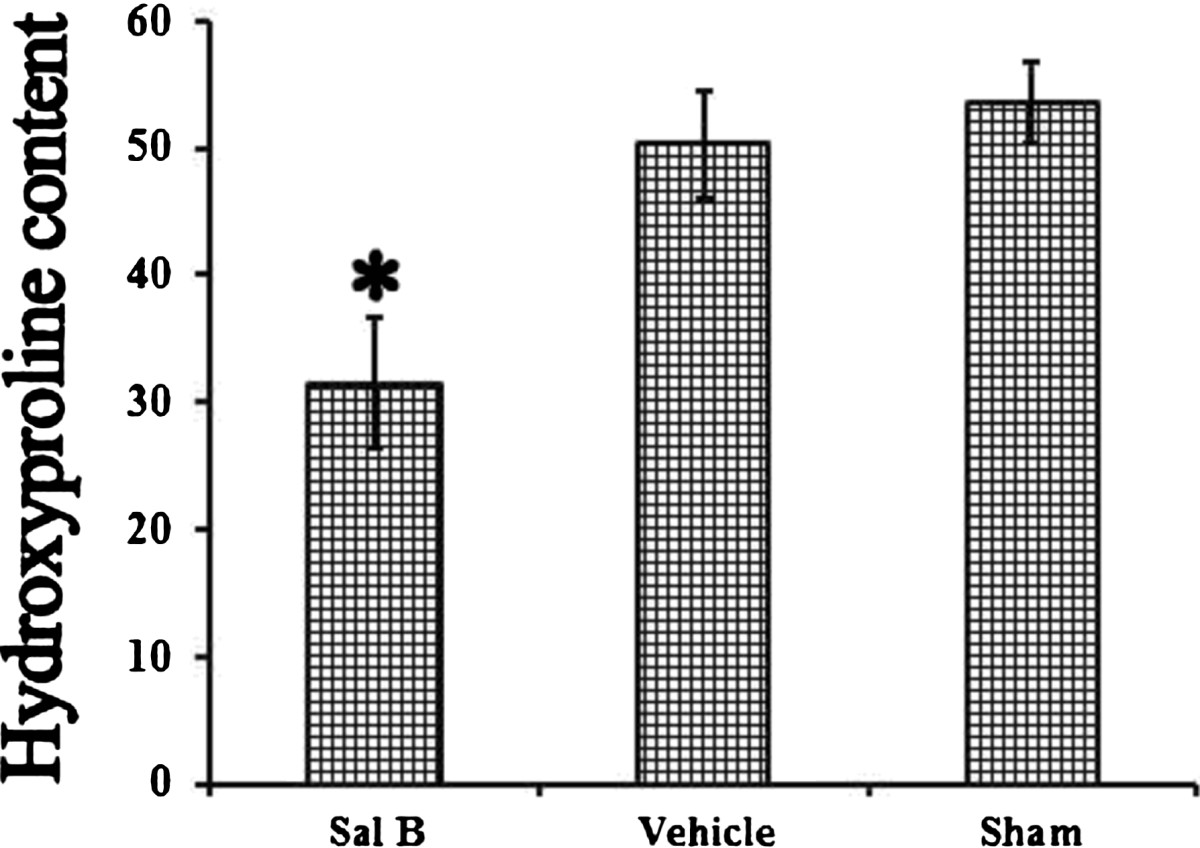
**Hydroxyproline content was evaluated.** Hydroxyproline levels are expressed as the mean ± standard deviation of hygrotissue. The Sal B group showed the lowest hydroxyproline level. *P < 0.05, compared with the other two groups.

As shown in Figure 1, HPC concentration in epidural scar tissue in the Sal B group (31.54 ± 5.06) was significant lower than that in the Vehicle group (50.34 ± 4.19, P = 0.006) and the Sham group (53.59 ± 3.27,P = 0.001). The content in Vehicle group showed no significant difference compared with the Sham group (P = 0.249).

### Histological analysis

**Figure 2:**
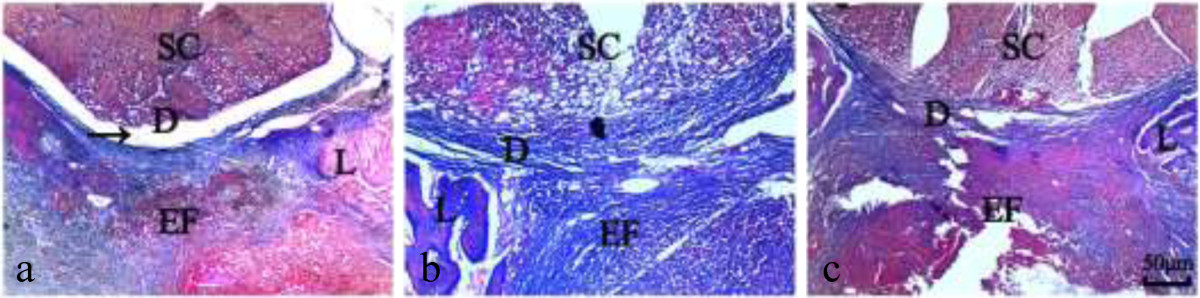
**Masson’s trichrome staining for epidural adhesion in the laminectomy sites treated with Sal B (a), Vehicle (b) or nothing (c). a**: Loose scar tissues without adherence to the dura mater were seen in the Sal B group. **b**, **c**: Dense scar tissues adhered to the dura mater were observed in the Vehicle and Sham groups. The magnification was 100×. SC = spinal cord, D = dura mater, EF = epidural fibrosis, L = laminectomy.

**Figure 3:**
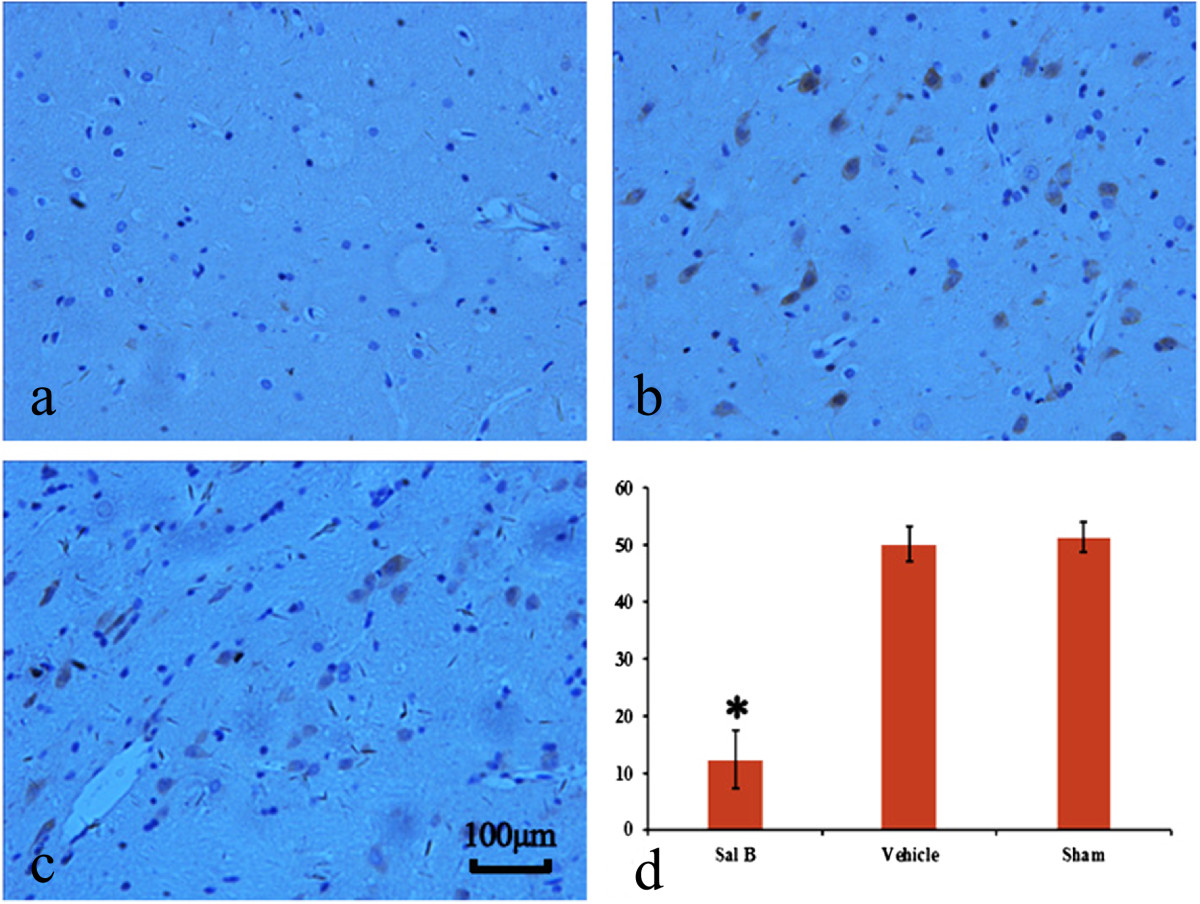
**Effect of Sal B on vascular endothelial growth factor (VEGF).** Immunohistochemistry for VEGF in epidural scar tissues treated with Sal B **(a)**, Vehicle **(b)** or nothing **(c)**. As shown in statistical analysis **(d)**, the density of VEGF in the Sal B group **(a)** was less than the other 2 groups. *P < 0.05 vs the Vehicle and the Sham group. The density of VEGF in the Vehicle group was similar to that of the Sham group. The magnification was 400 ×.

In the Sal B group, loose or little epidural scar adhesion was seen (Figure 2a). In the Vehicle group and the Sham group, dense epidural scar tissue with widespread adhesions to the dura mater was observed (Figure 2b,c). The immunohistochemistry analysis for VEGF showed that less VEGF was observable in the Sal B group versus the Vehicle and Sham groups. Representative sections are shown in Figure 3.

### Effect of Sal B on fibroblasts and inflammatory cells

**Figure 4:**
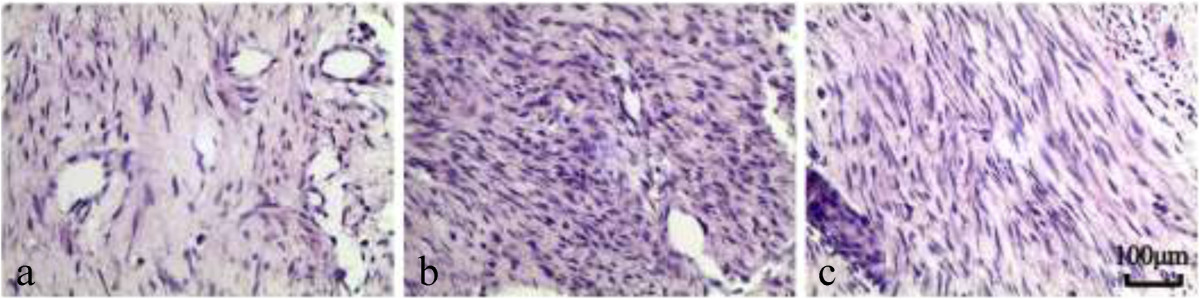
**H&E staining of fibroblasts in epidural scar tissues treated with Sal B (a), Vehicle (b) or nothing (c).** The density of fibroblasts in the Sal B group **(a)** was less than those of the other 2 groups. The density of fibroblasts in the Vehicle group **(b)** was similar to that of the Sham group **(c)**.

The fibroblasts and inflammatory density grades of epidural scar tissue in each group are listed in Table 3. The fibrotic and inflammatory cell densities in the Sal B group were lower than those of the Vehicle group and the Sham group. Both fibroblast and inflammatory cell densities were similar between the Vehicle group and the Sham group. Representative sections are shown in Figure 4.

**Table 3:** **Grades of fibroblast and inflammatory cell densities**

Groups	Fibroblast density	Inflammatory cell density
Sal B		
Sal B 1	1	1
Sal B 2	2	1
Sal B 3	1	1
Sal B 4	1	1
Sal B 5	1	2
Vehicle		
Vehicle 1	3	3
Vehicle 2	3	2
Vehicle 3	3	3
Vehicle 4	3	3
Vehicle 5	3	3
Sham		
Sham 1	3	3
Sham 2	3	3
Sham 3	3	3
Sham 4	3	3
Sham 5	3	3

### Effect of Sal B on IL-6 and TGF- β1

**Figure 5:**
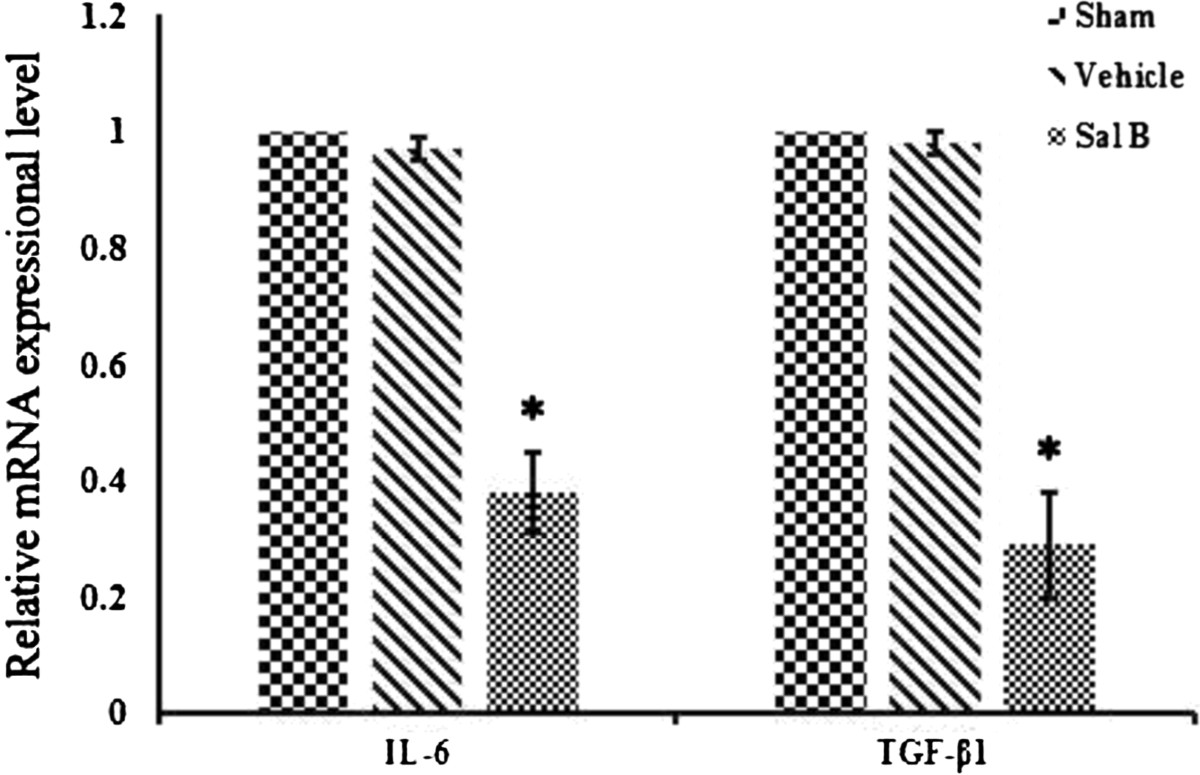
**mRNA expression of inflammatory factors (IL-6 and TGF-β1) in epidural scar tissue from each group.** Data from qPCR. *P < 0.05, compared with the Sham group.

The mRNA expression levels of TGF-β1 and IL-6 are shown in Figure 5. The Sal B group had significantly lower expression of both than the Vehicle group (P = 0.014) and the Sham group (P = 0.005). The Vehicle group and Sham group did not show a significant difference (P = 0.217).

## Discussion

Usually, epidural fibrosis forms after laminectomy, and discectomy leads to an unfavorable clinical outcome. It is caused by fibrous scar tissue hyperplasia, which could repair the local defect post-surgery. The fibroblast plays an important role in the repair process[7]. After being activated by inflammatory factors, it propagates and produces collagen fibers to act in a repairing role. However, local excessive inflammatory and fibrotic processes will cause excessive local collagen deposition. As a result, excessive scar tissue, transformed from fibrous connective tissue, can easily squeeze the dura mater or nerve root. Spinal stenosis, nerve root mobility restriction and dural compression could result. Therefore, it is widely accepted that EF is characterized by the accumulation of fibroblasts, the deposition of extracellular matrix proteins, and the distortion of normal tissue architecture with inflammation[18]. To effectively curtail EF, it is important to reduce these three factors[4].

In the present study, the potential ability of Sal B to reduce epidural scar adhesion and inhibit fibrotic and inflammatory cell proliferation at laminectomy sites was suggested. Multiple evaluations, including the Rydell classification, histological analysis, hydroxyproline content, VEGF density grade and qPCR suggested Sal B effectively prevents EF in laminectomized rats. The anti-fibrotic, anti-inflammatory, and anti-proliferative properties of Sal B have been used in different medical fields[14, 16, 17]. The present study confirms its beneficial properties in laminectomized rats. One effect of Sal B that reduced fibrosis was its downregulation of IL-6 and TGF-β1, which are suggested to play important roles in the promotion and/or development of EF[4–7]. Previous and current data may explain some if not all of the possible mechanisms that make Sal B effective in curtailing EF[20–22]. We hypothesize that the mechanism by which Sal B inhibits EF is its reduction of inflammatory cytokines, fibroblast proliferation and collagen deposition by blocking several signaling pathways, such as NF-κB and ERK signaling[16, 23, 24].

Recently, different medication have been proved to play curative effect on preventing EF. Immunosuppressive agent, antineoplastic agent and antibiotics all supported a good effectiveness on the prevention of EF[25–27]. To avoid the complication from the high-dose agents and find an optimal dose, we believe a combination between the aforementioned agent and Sal B will be a good strategy. More research will be performed. To our knowledge, this is the first study to suggest that Sal B prevents EF by down-regulating inflammatory activity and reducing hydroxyproline deposition in rats. In the present study in EF rats, we did not attempt to define the most effective dose or high-dose toxicity. Undoubtedly, more research on the safety, long-term effects, safe effective concentration, and possible side effects and adverse effects of Sal B are all warranted before clinical trials and application.

## Conclusions

Sal B can inhibit fibroblast proliferation, blood vessel regeneration, and inflammatory factor expression. Thus, Sal B is able to prevent epidural scar adhesion in post-laminectomy rats.

## Electronic supplementary material

Additional file 1:ARRIVE guideline.(DOCX 27 KB)

Below are the links to the authors’ original submitted files for images.

Authors’ original file for figure 1

Authors’ original file for figure 2

Authors’ original file for figure 3

Authors’ original file for figure 4

Authors’ original file for figure 5

## Abbreviations

EF: Epidural fibrosis
Sal B: Salvianolic acid B
VEGF: Vascular endothelial growth factor
IL-6: Interleukin-6
TGF-β1: Transforming growth factor-β1.
