# Supplementary material for: Study on salvianolic acid B in the reduction of epidural fibrosis in laminectomy rats
Source: BMC Musculoskelet Disord. 2014 Oct 7;15:337. doi: 10.1186/1471-2474-15-337 (PMC4289272; doi:10.1186/1471-2474-15-337)
Supplement: Supplementary file 1 — Additional file 1:ARRIVE guideline.(DOCX 27 KB) [file 12891_2014_2372_MOESM1_ESM.docx]

**ARRIVE guideline**

|  | **ITEM** | **RECOMMENDATION** |
| --- | --- | --- |
| Title | 1 | Study on Salvianolic acid B in the reduction of epidural fibrosis in laminectomy rats |
| Abstract | 2 | **Background:** Epidural fibrosis (EF) is a common complication after laminectomy. Salvianolic acid B (Sal B) is a major bioactive component of a traditional Chinese medical agent, Salvia miltiorrhiza, which has shown anti-inflammatory, anti-fibrotic and anti-proliferative properties.  **Objection:** The object of this study was to investigate the effect of Sal B on the prevention of epidural fibrosis in laminectomy rats. A controlled double-blinded study was conducted in sixty healthy adult Wistar rats.  **Methods:** All rats that underwent laminectomy at the L1-L2 levels. The rats were randomly divided into 3 groups of 20: (1) Sal B treatment group; (2) Vehicle group; (3) Sham group (laminectomy without treatment). All rats were sacrificed 4 weeks post-operatively. The extent of epidural fibrosis, fibroblast proliferation and the expression of vascular endothelial growth factor (VEGF) and inflammatory factors were analyzed.  **Results:** The recovery of all rats was uneventful. In the laminectomy sites treated with Sal B, the dura mater showed no adhesion. Collagen deposition was significantly lower in the Sal B group than the other two groups. In addition, both fibroblast and inflammatory cell counting in the laminectomy sites treated with Sal B showed better grades than the other two groups. The expression of VEGF and inflammatory factors in operative sites also suggested better results in the Sal B group than the other two groups.  **Conclusions:** Sal B inhibits fibroblast proliferation, blood vessel regeneration, and inflammatory factor expression. Thus, Sal B is able to prevent epidural scar adhesion in post-laminectomy rats. |
| **INTRODUCTION** | | |
| Background | 3 | 1. Failed back surgery syndrome (FBSS) is gaining attention from both lumbar surgeons and lumbar laminectomy patients. FBSS occurs in 8-40% of patients who undergo lumbar disc surgery [1]. Recurrent persistent low back pain and chronic nerve radicular are the main characteristics for FBSS [2]. Epidural fibrosis (EF) is widely accepted to be the main contributor to FBSS [3-5]. In the present research employing laminectomy rats to simulate post lumbar surgery patient, the efficacy of Sal B in the prevention of EF was evaluated. 2. The laminectomy rat model applied in the present study was reported in several previous researches [4-5]. It is widely accepted by researchers all over the world [6-8].   In summary, the surgery was performed under sterile conditions with basic surgical tools. All the rats were numbered individually after being restrained on a warm pad in the prone position. The fur of each rat was shaved around L1 and L2 and the exposed skin was sterilized. After that L1-L2 total laminectomy was performed with a micro-rongeur. Close attention was paid not to traumatize the dura and the nerve roots. After complete hemostasis with saline, the wound site was surgically closed. |
| Objectives | 4 | 1. Sal B can inhibit fibroblast proliferation, blood vessel regeneration, and inflammatory factor expression. 2. Sal B is able to prevent epidural scar adhesion in post-laminectomy rats. |
| **METHODS** | | |
| Ethical statement | 5 | The present research was performed in compliance with the principles of International Laboratory Animal Care and with the European Communities Council Directive (86/809 /EEC). |
| Study design | 6 | For each experiment, give brief details of the study design including:   1. Animals were randomly divided into three groups (20 rats in each group): 1) Sal B treatment group (Sal B 30 mg/kg, diluted in saline); 2) Vehicle group (saline); 3) Sham group (laminectomy without treatment). 2. **Randomization procedure:** the rats were divided randomly with random number table method.   **Assessing results:** The assistants who performed the treatment intervention were blinded on the agentia they used. The other group of assistant who performed Rydell assessment were blinded on the group of rats. |
| Experimental procedures | 7 | For each experiment and each experimental group, including controls, provide precise details of all procedures carried out.  For example:   1. Sal B (purity > 98%) was purchased from the National Institute for the Control of Pharmaceutical and Biological Products (Beijing, China). Sal B (30 mg/kg, diluted in saline) was employed. In the Sal B treatment group, salvianolic acid B (30 mg/kg, diluted in saline) was administered intragastrically. In the Vehicle group, the same volume of saline was given in the same way. In the Sham group, no special treatment was performed post-laminectomy.   Anesthetization was performed by intra-peritoneal injection of 10% chloral hydrate (0.3 ml/100 g body weight).  Euthanasia was performed with an overdose of aforementioned narcotic.  The main equipment employed in the present research were list with detailed supplier as following:  The light microscope (Olympus ix-71)  The micro-rongeur (FST 16021-14、FST16221-14, Germen)  Quantitative real-time PCR (qPCR) was performed using the Bio-Rad MYIQ2 (USA)   1. Intervention was performed every morning of the day from 8:30 to 11:00. 2. Animals were housed in the local laboratory located in Qingdao University Medical College. 3. The anesthetization employed in the present research was reported in the previous studies [9-11]. |
| Experimental animals | 8 | 1. Wistar rats, adult healthy male, weight 250±20g, age 56±4d. 2. Wistra rat is a kind of experimental animal widely accepted all over the world. |
| Housing and husbandry | 9 | Provide details of:   1. **Housing**: specific pathogen free [SPF];   **Type of cage or housing**: pre-surgery feeding cage sized 50cm×80cm; post-surgery feeding cage sized 20cm×30cm.  **Bedding material**: pieces of wood;  **Number of cage companions:** pre-surgery: every 5 rats were housed in one cage; post-surgery: every single rat were housed in one cage.   1. Animals were housed in the local laboratory under the conditions of 20 to 25°C room temperature, a 12 hour light-dark cycle and clean food and water ad libitum. 2. All efforts were made to minimize animal numbers and suffering. The rats were housed for 10 days to adjust them to the environment pre-operatively. |
| Sample size | 10 | 1. Sixty rats were involved in the present research.   All rats were divided into three groups, with 20 rats in each group.   1. In every group with 20 rats:   5 rats were used for Macroscopic assessment.  5 rats were used for Determination of Hydroxyproline content (HPC) analysis.  5 rats were used for Histological analysis.  5 rats were used for analyzing IL-6 and TGF-β1 Concentrations.   1. In the present research, with 5 rats being involved in each verified step, there is no need to do the independent replications of each experiment. |
| Allocating animals to experimental groups | 11 | 1. Sixty rats were randomly divided into 3 groups with random number table method. 2. Randomly, rat was individually numbered after dividing. The evaluations were performed in order. |
| Experimental outcomes | 12 | **Rydell classification** (grade 0=epidural scar tissue was not adherent to the dura mater; grade 1=epidural scar tissue was adherent to the dura mater, but easily dissected; grade 2=epidural scar tissue was adherent to the dura mater, and it was difficult to dissect without disrupting the dura matter; grade 3=epidural scar tissue was firmly adherent to the dura mater and could not be dissected).  **The number of fibroblasts and inflammatory cells were calculated based on a previous study [5]**: grade 1, fewer than 100 fibroblasts/inflammatory cells per ×400 field; grade 2, 100 to 150 fibroblasts/inflammatory cells per ×400 field; grade 3, more than 150 fibroblasts/inflammatory cells per ×400 field. Three different counting areas were selected at the middle and at the margins of the laminectomy sites. The cells were counted and their mean was calculated. |
| Statistical methods | 13 | 1. **Single-factor analysis of variance (ANOVA)**:   Macroscopic assessment of epidural scar adhesion.  Hydroxyproline content (HPC).  Histological analysis.  **q-test**  mRNA expression of inflammatory factors (IL-6 and TGF-β1) in epidural scar tissue.   1. **Unit:** **1**   Macroscopic assessment of epidural scar adhesion  Hydroxyproline content (HPC).  mRNA expression of inflammatory factors (IL-6 and TGF-β1) in epidural scar tissue.  VEGF density.   1. After assessing the data with single-factor analysis of variance (ANOVA) and the q-test, the data met the assumptions of the statistical approach. |
| **RESULTS** | | |
| Baseline data | 14 | Weight change in the period of 38 days:  Rats in the Sal B group changed from 248±19g to 267±15g  Rats in the Vehicle group changed from 251±20g to 258±13g  Rats in the Sham group changed from 248±18g to 268±19g |
| Numbers analysed | 15 | Macroscopic assessment: 5/20  Determination of Hydroxyproline content (HPC) analysis: 5/20  Histological analysis: 5/20  Analyzing IL-6 and TGF-β1 Concentrations: 5/20 |
| Outcomes and estimation | 16 | Statistical significance was assumed at p < 0.05. |
| Adverse events | 17 | 1. The recovery of all rats was uneventful. All rats showed no sign of wound infection, neurological deficit or disturbance of wound healing. 2. No modifications to the experimental protocols for reducing adverse events. |
| **Discussion** |  |  |
| Interpretation/scientific implications | 18 | 1. The results of the present research were discussed with taking into account the study objectives and hypotheses, current theory and other relevant studies in the literature. 2. Comment on the study limitations were given out in the last paragraph of the present manuscript. 3. All efforts were made to minimize animal numbers and suffering. The strict aseptic and avoiding unnecessary trauma during surgery played an important role in curtailing the post-operative infection. Thus, all the rats of the present study could show no sign of wound infection or dead. |
| Generalisability/translation | 19 | Comments were given out in the discussion section on whether, and how, the findings of this study are likely to translate to other species or systems, including any relevance to human biology. |
| Funding | 20 | All funding sources (including grant number) and the role of the funder(s) were given out in the present study. |

**Reference**

1. Burton CV, Kirkaldy-Willis WH, Yong-Hing K, Heithoff KB: **Causes of failure of surgery on the lumbar spine.** *Clinical Orthopaedics and Related Research* 1981, **157**: 191-199.

2. Guyer RD, Patterson M, Ohnmeiss DD: **Failed back surgery syndrome: diagnostic evaluation.** *The Journal of the American Academy of Orthopaedic Surgeons* 2006, **14**: 534-543.

3. Siqueira EB, Kranzler LI, Dharkar DD: **Fibrosis ofthe dura mater: a cause of “failed back” syndrome.** *Surgical Neurology* 1983, **19**:168–170.

4. Zhang C, Kong X, Liu C, Liang Z, Zhao H, Tong W, Ning G, Shen W, Yao L, Feng S: **ERK2 small interfering RNAs prevent epidural fibrosis via the efficient inhibition of collagen expression and inflammation in laminectomy rats**. *Biochem Biophys Res Commun* 2014, **444**:395-400.

5. Zhang C, Kong X, Zhou H, Liu C, Zhao X, Zhou X, Su Y, Sharma HS, Feng S: **An Experimental Novel Study: Angelica sinensis Prevents Epidural Fibrosis in Laminectomy Rats via Downregulation of Hydroxyproline, IL-6, and TGF- β 1.** *Evid Based Complement Alternat Med* 2013 2013;2013:291814. doi: 10.1155/2013/291814.

6. Wang Z, Wang Y, Xie P, Liu W, Zhang S：**Calcium channel blockers in reduction of epidural fibrosis and dural adhesions in laminectomy rats.** *Eur J Orthop Surg Traumatol* 2013, 2013 Dec 24. [Epub ahead of print].

7. Zhang C, Kong X, Ning G, Liang Z, Qu T, Chen F, Cao D, Wang T, Sharma HS, Feng S: **All-trans retinoic acid prevents epidural fibrosis through NF-κB signaling pathway in post-laminectomy rats.** *Neuropharmacology* 2014, **79**:275-81.

8. Sandoval MA, Hernandez-Vaquero D: **Preventing peridural fibrosis with nonsteroidal anti-inflammatory drugs.** *European Spine Journal* 2008, **17**:451-455.

9. Lubina ZI, Baranovic S, Karlak I, Novacic K, Potocki-Karacic T, Lovrić D: **The grading model for the assessment of the total amount of epidural fibrosis in postoperative lumbar spine.** *European Spine Journal* 2013 **22**:892-897.

10. Cicuendez M, Munarriz PM, Castaño-Leon AM, Paredes I: **Dorsal myelopathy secondary to epidural fibrous scar tissue around a spinal cord stimulation electrode.** *J Neurosurg Spine* 2012 **17**:598-601.

11. Kasimcan MO, Bakar B, Aktaş S, Alhan A, Yilmaz M: **Effectiveness of the biophysical barriers on the peridural fibrosis of a postlaminectomy rat model: an experimental research.** *Injury* 2011 **42**:778-781.
