# Supplementary figures and images for: Study on salvianolic acid B in the reduction of epidural fibrosis in laminectomy rats
Source: BMC Musculoskelet Disord. 2014 Oct 7;15:337. doi: 10.1186/1471-2474-15-337 (PMC4289272; doi:10.1186/1471-2474-15-337)

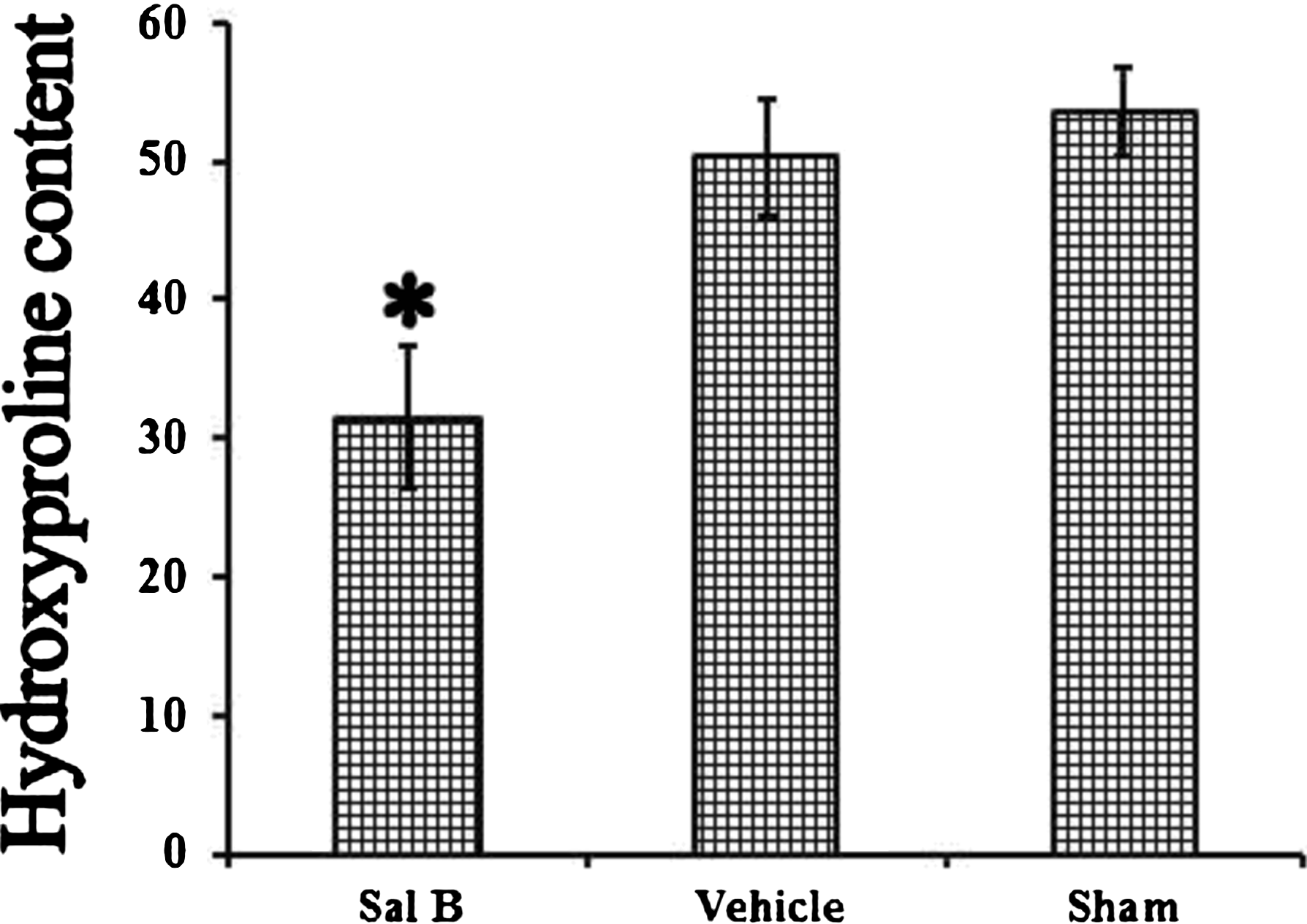

Supplement: Supplementary file 2 — Authors’ original file for figure 1 [file 12891_2014_2372_MOESM2_ESM.tif]

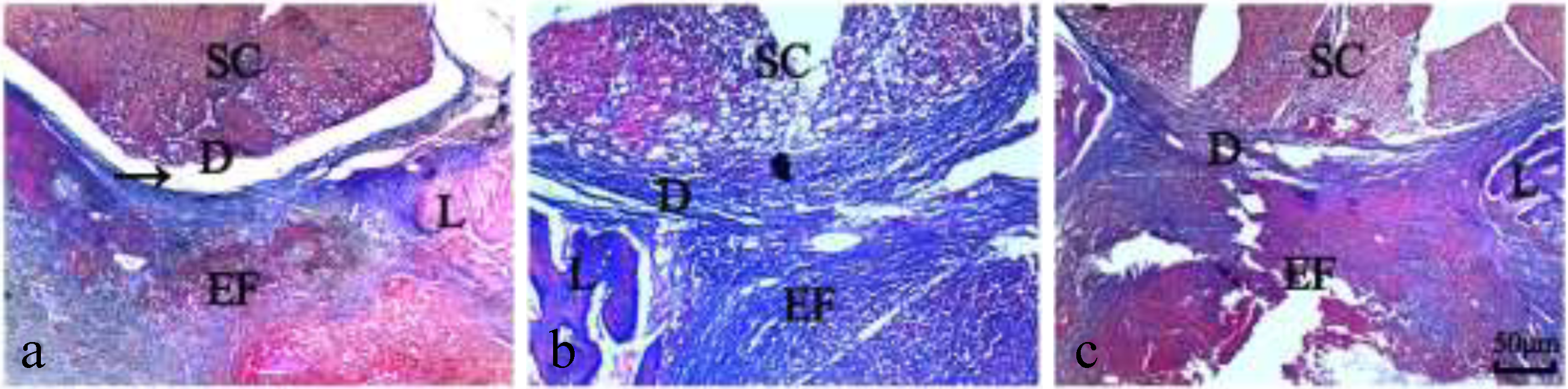

Supplement: Supplementary file 3 — Authors’ original file for figure 2 [file 12891_2014_2372_MOESM3_ESM.tif]

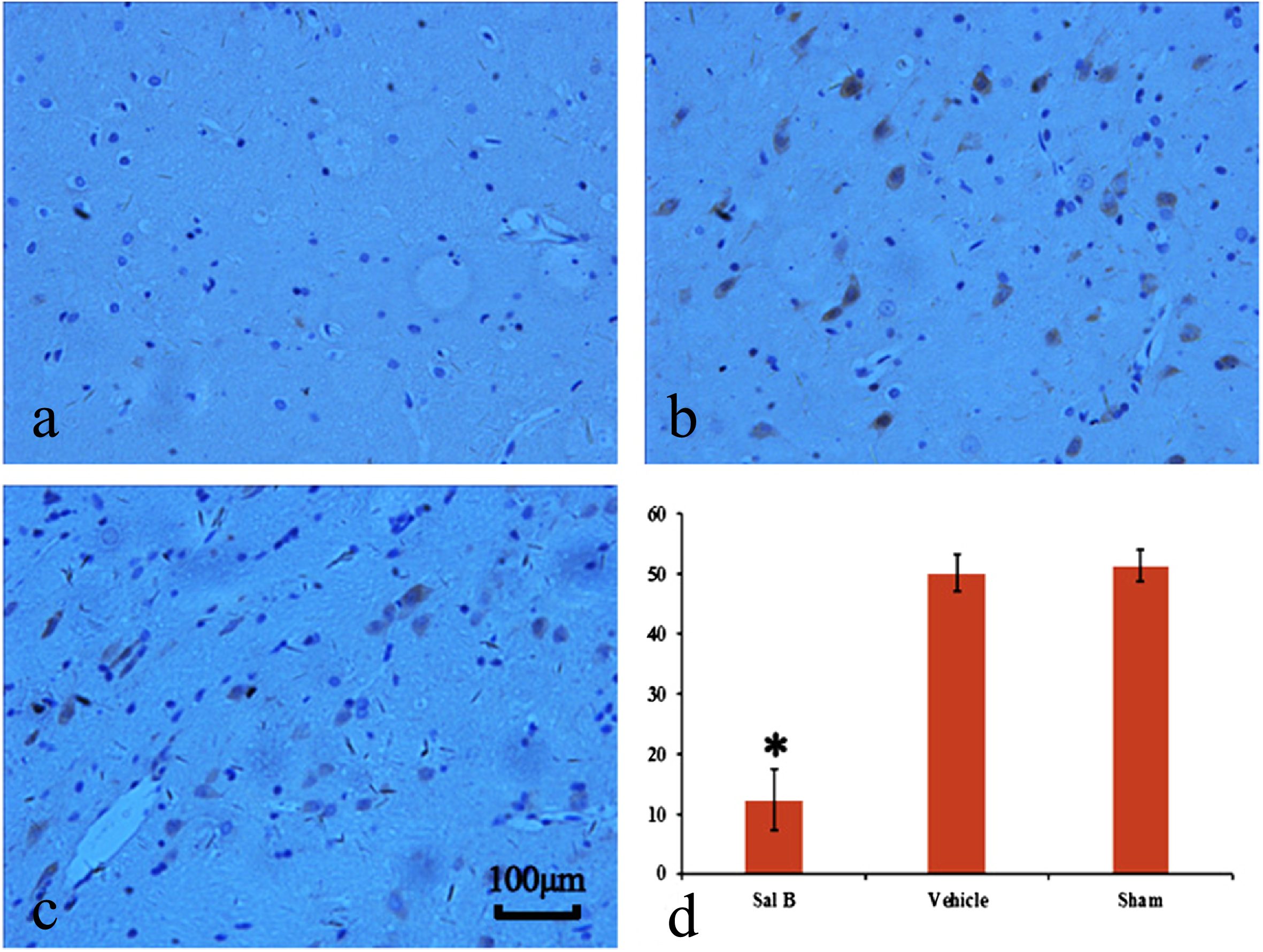

Supplement: Supplementary file 4 — Authors’ original file for figure 3 [file 12891_2014_2372_MOESM4_ESM.tiff]

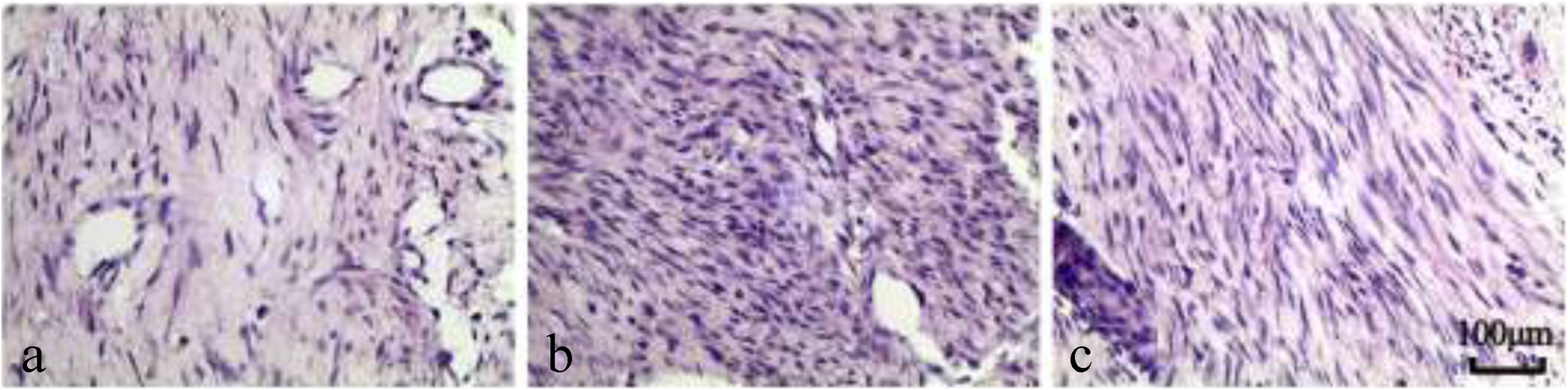

Supplement: Supplementary file 5 — Authors’ original file for figure 4 [file 12891_2014_2372_MOESM5_ESM.tif]

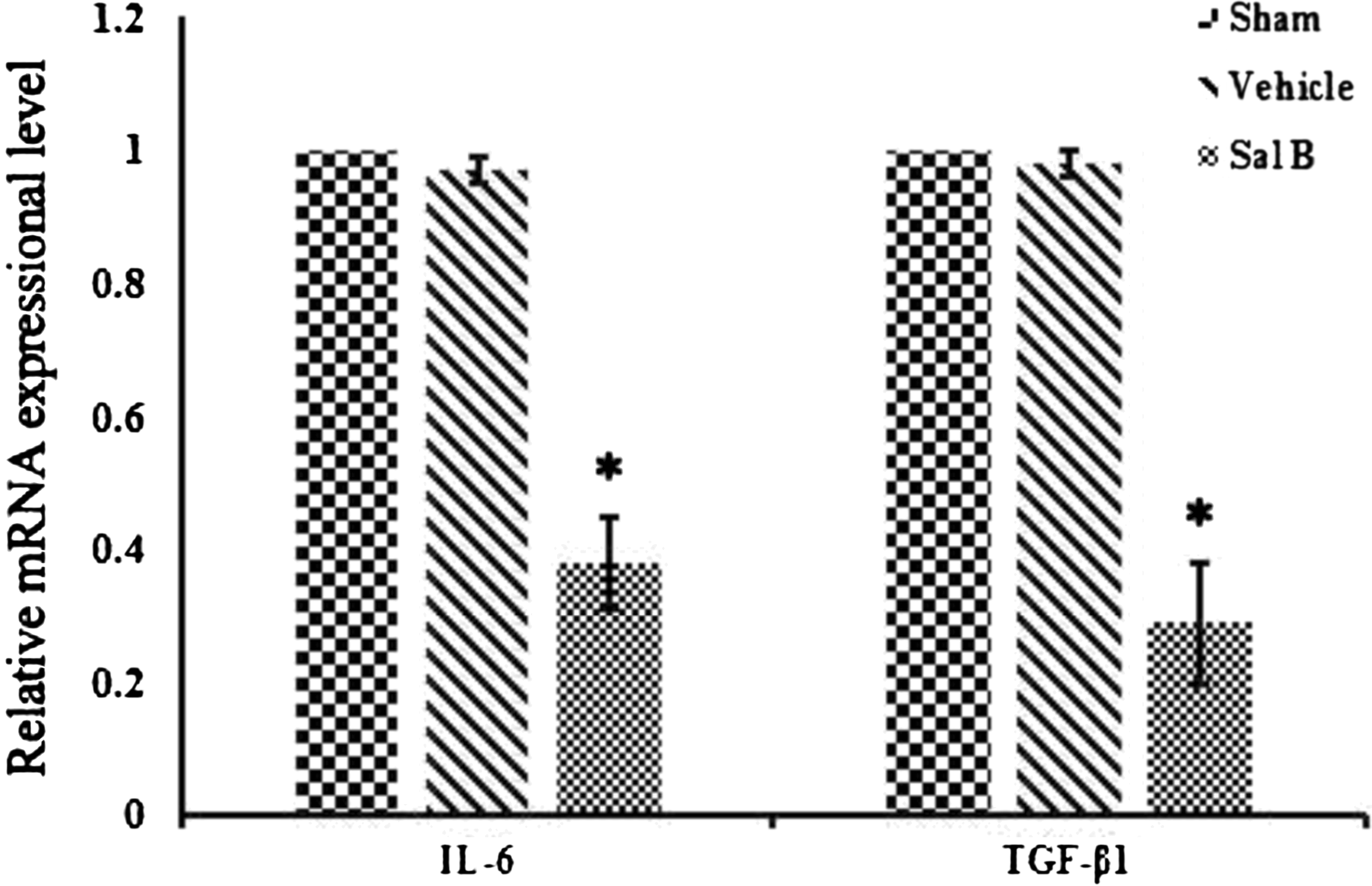

Supplement: Supplementary file 6 — Authors’ original file for figure 5 [file 12891_2014_2372_MOESM6_ESM.tif]
